# Multimodal Physiological Responses During Sentence Recognition at Individualized Speech-in-Noise Thresholds

**DOI:** 10.3390/s26206613

**Published:** 2026-10-19

**Authors:** Shuang Qi, Linda M. Thibodeau

**Affiliations:** 1 Department of Speech, Language and Hearing Sciences, The University of Texas at Dallas, Richardson, TX 75080, USA; 2 Callier Center for Communication Disorders, Richardson, TX 75080, USA

**Keywords:** electrodermal activity, listening effort, photoplethysmography, pupil diameter, speech in noise

## Abstract

By individualizing speech-in-noise level, one can aim to compare physiological responses near a comparable recognition level across listeners with different hearing abilities. We examined whether the aim was realized and whether pupil diameter, skin conductance, and photoplethysmography-derived heartrate provided usable responses during sentence recognition. Thirty-three young adults with normal or near-normal audiograms repeated sentences in multitalker babble at individually calibrated levels. Code audit identified the calibration estimate as a model-based whole-sentence SNR50, rather than the intended SNR75. Actual whole-sentence accuracy averaged 43.6% (ranging from 20% to 70%). Relative to a 500 ms pre-speech reference, mean pupil change was 0.17 mm during encoding–retention (95% CI [0.13, 0.21]; n = 19) and 0.14 mm during verbal response ([0.08, 0.20]; n = 13). Corresponding skin-conductance changes were 0.02 μS ([0.01, 0.04]; n = 30) and 0.07 μS ([0.04, 0.09]; n = 30). Skin conductance was greater during verbal response, an interval also containing speech production, respiration, and movement; this contrast cannot isolate listening-related activity. No cross-modal or physiology–behavior association survived false-discovery-rate correction. The short reference did not support heart-rate change estimation. Individual calibration provided an approximate performance anchor but did not verify equivalent observed performance or effort. The data describe stage-related pupil and conductance reactivity but cannot rank modalities by sensitivity to changing listening demand.

## 1. Introduction

Individuals often rely on top-down cognitive processing and executive functioning to “fill in the blanks” of a degraded auditory signal [1,2]. However, standard clinical assessments of behavioral hearing (i.e., audiogram or speech-in-noise recognition scores) may fail to capture the active neural mechanics of real-life communication [3,4]. Consequently, people may maintain high speech intelligibility scores with a high cognitive cost [5,6,7], or listening effort, the cognitive resources actively deployed to understand speech [8].

Listening effort is multi-dimensional, reflecting variances in task demand, autonomic arousal, and resource allocation [9,10]. Table 1 summarizes the main subjective, behavioral and physiological measures used in listening-effort research, together with their major advantages and limitations. Self-reported listening-effort measures (i.e., questionnaires) often reflect a listener’s subjective frustration rather than objective physiological load [11]. Pupil diameter, electrodermal activity (EDA), and cardiovascular signals are three physiological measures used in listening-effort research; none is specific to listening effort. Pupil size varies with cognitive demand, arousal, attention, orienting, and luminance [12,13]. Skin conductance (EDA metric) reflects eccrine sympathetic activation, but it can also respond to novelty, emotion, movement, and events in preceding task epochs [14,15]. Heart rate (HR) combines autonomic, respiratory, motor, and postural influences [14,16]. Concurrent recording may characterize task-related physiological patterns, but the cognitive cause of a change requires appropriate controls [9,10,17].

Signal-to-noise ratio (SNR) specifies an acoustic condition, recognition accuracy is a behavioral outcome, and effort is resource allocation, which might be altered by motivation, available capacity, and task goals at a given accuracy [5,6,8,23]. At a fixed SNR, listeners with different speech-in-noise abilities may perform at different levels. Prior lab testing in young adults with self-reported typical hearing showed that listening effort was only significantly correlated with poor digits-in-noise scores at a medium-difficulty (–12 dB) SNR, but not at easier (–9 dB) or harder (–15 dB) SNRs [24]. This finding aligns with the Framework for Understanding Effortful Listening, which posits that listening effort is a dynamic function of both task demand and listener motivation over time [8]. When a listening task is too simple, minimal cognitive resources are required; when a task is overwhelmingly difficult, listeners disengage and deploy fewer cognitive resources. Therefore, evaluating listening effort near an individualized, intermediate recognition level may help avoid conditions that are either too easy or sufficiently difficult to promote disengagement. Calibrating SNR to an individual psychometric target (i.e., SNR to perform 75% speech recognition accuracy) offers a possible performance anchor, but the short estimate has uncertainty and must be checked against performance in the later task. Equal target probabilities do not establish equal observed intelligibility, task demand, or effort.

Individualized recognition levels and concurrent physiological measurements have been examined previously. Adaptive threshold studies have related pupil response to intelligibility [25]; others have measured skin conductance and cardiac responses after equalizing recognition across hearing groups [26]. Keur-Huizinga et al. examined pupil, cardiovascular, and skin-conductance responses across three individualized intelligibility levels and related them to hearing acuity [21]. Plain et al. combined pupil and cardiovascular features at individualized levels [27], and Polo et al. evaluated a broader adaptive multimodal protocol [28]. These studies show why one calibrated condition cannot establish which sensor is most sensitive to changes in demand. The present contribution is a transparent evaluation of three concurrently recorded metrics, calibration transfer, stage-specific retention, and associations with hearing-related measures in this particular dataset.

We asked four exploratory questions. First, how closely did observed whole-sentence accuracy in the physiological task approach the intended common recognition anchor? Second, during that individually calibrated condition, were pupil and skin-conductance stage means different from a local pre-speech reference, and was photoplethysmography (PPG)-derived HR sufficiently supported for the same analysis? Third, did usable modalities covary across participants? Fourth, were stage responses associated with model-based SNR50, achieved whole-sentence accuracy, or general self-reported hearing? These outcomes address feasibility and convergent patterns, not comparative sensitivity to effort. Demand was not manipulated during physiological recording, no concurrent effort rating was obtained, and the verbal-response stage included speech production. We therefore present exploratory questions rather than confirmatory directional hypotheses.

## 2. Methods

### 2.1. Study Design and Interpretive Scope

This observational laboratory study examined within-participant physiological changes across auditory encoding plus retention and verbal response during one sentence-recognition condition. Individual calibration was intended to bring listeners near the same whole-sentence recognition probability. The physiological experiment should therefore be interpreted as testing participants at an individually estimated psychometric midpoint, rather than as testing participants under experimentally verified equivalent intelligibility conditions. Achieved accuracy in the later task was checked separately. Demand was not manipulated during physiological recording, and there was no listening-only or matched speech-production control. Physiological outcomes are described as task-evoked responses rather than process-pure measures of listening effort.

### 2.2. Participants and Ethics

**Figure 1:**
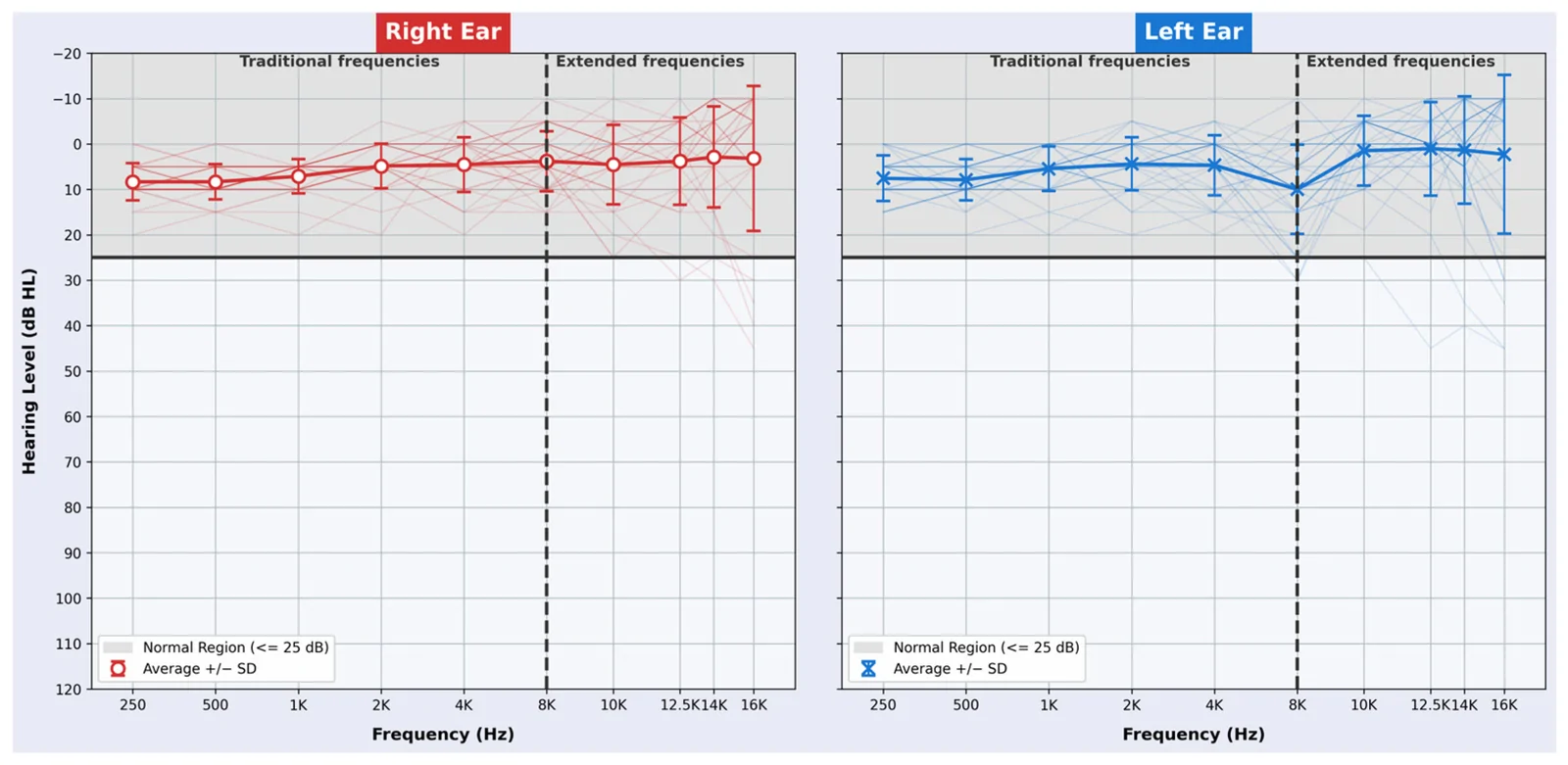
Pure-tone audiometry across traditional and extended high frequencies. Note. Right (red circles) and left (blue crosses) ear thresholds are displayed in separate panels. Faint traces represent individual participant profiles, while bold lines denote the cohort averages with standard deviation (±SD) error bars. The shaded gray region (≤25 dB HL) indicates the clinically normal hearing range. A dashed vertical line at 8 kHz separates traditional pure-tone audiometry from extended high-frequency testing.

Thirty-three volunteers aged 18–24 years (*M* = 20.55, *SD* = 1.72; 20 female, 12 male, and 1 preferred not to say) participated. The parent online study enrolled 321 participants through the university research platform. All members of that cohort were invited by email to this laboratory study, and the present sample comprised those who responded and completed testing during the six-month recruitment window. No participant was deliberately selected from the cohort, but participation depended on response to the invitation. All participants self-reported bilateral normal hearing, acquired English before age six, and reported no history of neurological or psychological disorders. Pure-tone thresholds were measured from 0.25 to 16 kHz using a Grason-Stadler AudioStar Pro audiometer (Grason-Stadler, Eden Prairie, MN, USA) and TDH-39 headphones (Telephonics, Farmingdale, NY, USA) in a sound booth. As shown in Figure 1, thirty-one participants had bilateral thresholds no greater than 25 dB HL from 0.25 to 8 kHz. Two had a unilateral 30 dB HL threshold at 8 kHz and were retained given that this elevation was only present at a single high frequency in one ear and the present study focused on within-subject task-evoked responses. The sample is therefore described as having clinically normal or near-normal audiometric thresholds. The University of Texas at Dallas Institutional Review Board approved the study (IRB-24-173, 30 September 2024), and all participants provided written consent.

### 2.3. General Procedures

After audiometry, participants completed the adaptive sentence-in-noise calibration to establish the individualized SNR level (SNR50). They then completed a separate 20-sentence recognition task at a participant-specific SNR while pupil diameter, skin conductance, and photoplethysmography (PPG) were recorded.

### 2.4. Measures

#### 2.4.1. Model-Based Whole-Sentence SNR50

Before physiological recording, an individualized SNR was estimated from 20 sentence trials using an adaptive Bayesian procedure [29] implemented in Python 3.9.19 through PsychoPy 2024.2.5. Twenty root mean square (RMS) normalized hearing-in-noise test (HINT) sentences from lists 1 and 2 [30] were mixed with multitalker babble and presented in the free field. Babble was held at 65 dBA as measured at the participant’s head, and sentence RMS was varied trial by trial. Stimuli were delivered from 0 degrees azimuth at a nominal 30 cm distance through the loudspeaker used with the laboratory’s manikin head (Knowles Electronics Manikin for Acoustic Research). The experimenter scored a response as correct only when the entire sentence was repeated correctly. The study was designed as an SNR75 calibration, but code review showed that the target-probability variable was not used in the likelihood or stimulus-selection rule. The implemented model therefore estimated the 50% whole-sentence recognition midpoint, reported here as model-based SNR50. The final posterior mean after trial 20 was used directly as the presentation SNR in the physiological task. The model equation, individual results and audit are reported in Appendix A.

#### 2.4.2. Formal Speech-Recognition Task at SNR50

Participants completed 20 HINT sentences from lists 7 and 8 at their individualized SNR50 while pupil diameter, skin conductance, and PPG were recorded. The sentences were presented in random order. Whole-sentence accuracy was defined as the number of full correctly recognized sentences divided by 20. This criterion matches the all-words-correct rule used during SNR50 calibration.

**Figure 2:**
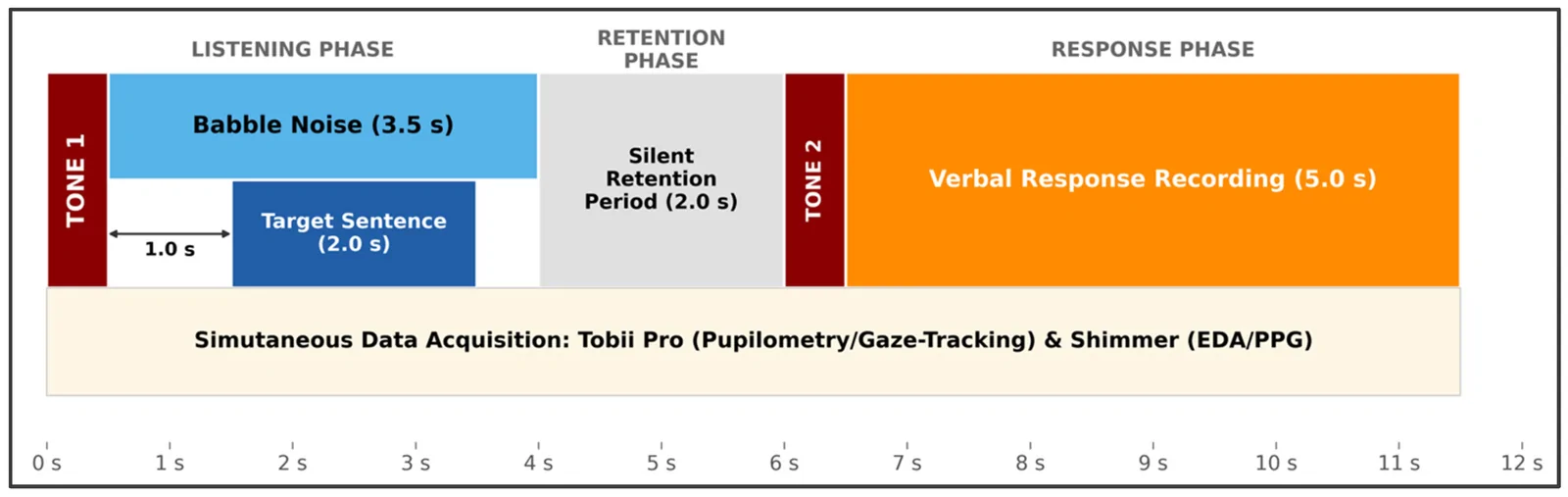
Nominal trial sequence for sentence recognition in babble. Note. The diagram is a schematic; analysis windows used recorded software event times and reconstructed target-speech onset, not the nominal clock. The approximately 2–3 s period without speech after most verbal responses was not a verified physiological recovery interval. EDA = electrodermal activity, PPG = photoplethysmography.

Detailed procedures for each trial (total = 20) are presented in Figure 2. To balance the need for physiological recovery with the prevention of cognitive fatigue and vigilance decrement, a continuous trial paradigm was employed. Each trial was set to last 12 s. Following the stimulus presentation and retention phases, there was a 5 s verbal response window. Behavioral observations indicated that most participants completed their verbal responses within the first 2 to 3 s. The remaining 2 to 3 s of silence served as an implicit inter-trial interval to maintain continuous ecological engagement and minimize fatigue. All participants finished all trials in about 5 min.

Specifically, each trial began with a 0.5 s 1000 Hz alert tone, followed by 3.5 s of babble noise and a 2 s target sentence that started 1 s after the noise onset. The baseline used in this study is the 0.5 s window right after the onset of the continuous babble to isolate the task-induced response allocated to sentence processing from the abrupt auditory change elicited by the noise onset [13]. The mixed stimulus was followed by a nominal 2 s retention interval, a 0.5 s response cue (1000 Hz tone), and a nominal 5 s response recording. Throughout this entire sequence, physiological responses were continuously recorded and timestamped across all testing steps. Data were transmitted and recorded via hardware and software (i.e., Tobii Pro Eye Tracker Manager v2.7.4) connections in Python.

#### 2.4.3. Physiological Recording

Pupil diameter was recorded in millimeters at 120 Hz using a Tobii Pro Spectrum (Tobii AB, Stockholm, Sweden) after five-point gaze calibration (four corners and the center). EDA and PPG were recorded simultaneously with the eye tracker at 128 Hz with a Shimmer3 GSR+ (Shimmer Research, Dublin, Ireland) and its optical pulse-sensing input [31]. Two reusable dry metal Ag/AgCl finger electrodes contacted the medial phalanges of the index and middle fingers. The PPG probe was an ear clip. Both devices remained attached throughout physiological testing, but the Shimmer recording connection was stopped after each trial and re-established before the next trial to avoid prolonged-connection errors.

A fixation cross remained at the center of the screen during physiological recording. Participants sat in front of the eye-tracker screen and were instructed to remain seated, fixate the cross, and minimize movement. Testing took place in an air-conditioned laboratory maintained at approximately 74 degrees F (23 degrees C).

#### 2.4.4. Behavioral and Self-Report Variables

Behavioral variables include SNR50, whole-sentence accuracy in the 20-trial physiological task, and the mean 15-item Speech, Spatial and Qualities of Hearing Scale score (15iSSQ) [32]. The 15iSSQ was completed in the parent study from several days to six months before this study session. The 15iSSQ maintains the three-factor structure of the full form [33]: speech hearing (questions 1–5), spatial hearing (questions 6–10), and sound quality (questions 11–15). The score ranged from 0 (complete inability) to 10 (perfect ability), with higher scores indicating better self-reported hearing. The 15-item SSQ assesses perceived hearing abilities across a range of everyday situations rather than performance at a specific time point. Moreover, unlike the full SSQ [33], the 15-item version does not include items specifically addressing listening effort. It was therefore treated as a general self-report measure of hearing function. Given that all participants maintained clinically normal hearing across study phases, 15iSSQ scores were expected to remain relatively stable over the study period. Nevertheless, temporal variation in self-reported hearing ability cannot be ruled out.

### 2.5. Statistical Analysis and Power Considerations

#### 2.5.1. Statistical Analysis

The designed trial duration was 12 s; however, actual trials averaged around 14 s due to hardware latency and response variability. To correct for this, all physiological and eye-tracking signals were synchronized using empirical event timestamps rather than the planned schedule. Target-speech onset was estimated from the code and audio files, and the 500 ms babble-period interval immediately before that onset was the pre-speech reference, not a resting baseline. Physiological preprocessing and trial-support rules are specified in Appendix B [13,15,16,34,35].

Data analysis was conducted using Python (version 3.9.19) and the participant was treated as the independent unit. Trial features were averaged within participant; within-trial stage differences were averaged afterward. A participant entered a given modality-stage analysis with at least 14 of 20 valid trials. These are complete-case analyses within each required modality and stage; paired contrasts and correlations use the intersection of participants with valid component measures, not a common complete-case sample across all sensors. Six primary effects were tested: pupil and stage-level skin-conductance change against zero in each stage, plus response-minus-encoding–retention contrasts for each modality. Two-sided sign-flip tests used exact enumeration when n ≤ 20 and 50,000 random flips otherwise. The six *p* values formed one Benjamini–Hochberg false-discovery-rate (FDR) family. We report participant-bootstrap 95% confidence intervals (CIs) and Hedges’ gz. During peer-review reanalysis, we grouped the two pupil–skin-conductance associations, 12 physiology–behavior associations (2 modalities × 2 stages × 3 variables), three behavioral intercorrelations, and six trial-order slopes into separate families. These families were defined for the revision and were not prospectively preregistered. Associations used Spearman ρ, 20,000 participant bootstrap samples for CIs, and 50,000 label permutations for two-sided *p* values. Absolute HR was outside primary inference.

#### 2.5.2. Power Considerations

No a priori power analysis determined the convenience sample. We did not calculate observed post hoc power because it is determined by the observed *p* value and does not add evidence about a completed study [36,37]. Instead, achieved-sample sensitivity calculations used Fisher’s z approximation. At unadjusted two-sided *α* = 0.05 and 80% power, the minimum detectable absolute correlation was 0.47 at n = 33, 0.49 at n = 30, 0.60 at n = 19, 0.63 at n = 17, 0.71 at n = 13, and 0.73 at n = 12. Corresponding two-sided paired/one-sample t approximations yielded minimum detectable absolute dz values of 0.53 at n = 30, 0.68 at n = 19, and 0.85 at n = 13. These are planning/sensitivity approximations, not evidence that smaller effects are absent.

For prospective replication, 80% power at two-sided α = 0.05 would require approximately 85, 47, or 30 complete cases to detect |*ρ*| of 0.30, 0.40, or 0.50, respectively. Recruitment should allow for the modality-specific losses documented here.

## 3. Results

### 3.1. Behavioral Description

Across 33 participants, the model-based SNR50 estimates averaged 2.24 dB SNR (SD = 1.63 dB, 95% bootstrap CI [1.66, 2.76]; range −1.48 to 5.81). Whole-sentence accuracy averaged 0.44 (SD = 0.13, [0.39, 0.48]) and ranged from 0.20 to 0.70, corresponding to 4–14 fully correct sentences. Twenty of 33 participants scored between 40% and 60%. The estimates thus supplied a rough performance anchor, not experimentally verified equivalent intelligibility. The 15iSSQ averaged 8.50 (SD = 0.88, [8.19, 8.79]; range 6.67–10.00).

The 20-trial calibration retained substantial conditional uncertainty: mean final posterior SD was 1.85 dB, mean 95% credible-interval width was 7.25 dB, and the trial-15 to trial-20 estimate changed by 0.91 dB on average.

SNR50 was positively associated with subsequent whole-sentence accuracy (*ρ* = 0.37, 95% CI [−0.01, 0.68], *p* = 0.033, *q* = 0.05) and negatively with 15iSSQ (*ρ* = −0.37, [−0.67, 0.002], *p* = 0.035, *q* = 0.05). Whole-sentence accuracy and 15iSSQ were weakly related (*ρ* = −0.09, [−0.45, 0.30], *p* = 0.626, *q* = 0.63). However, none met the *q* < 0.05 criterion.

### 3.2. Data Retention and Physiological Stage Effects

All 33 participants had recordings for each modality, but valid trial and participant counts differed markedly by stage (Table A2). The dominant pupil losses were absent observed reference samples or inadequate stage support; Shimmer clock failures accounted for most EDA exclusions. Only 13 participants met the pupil response criterion. Eligible participants therefore differed across analyses.

**Figure 3:**
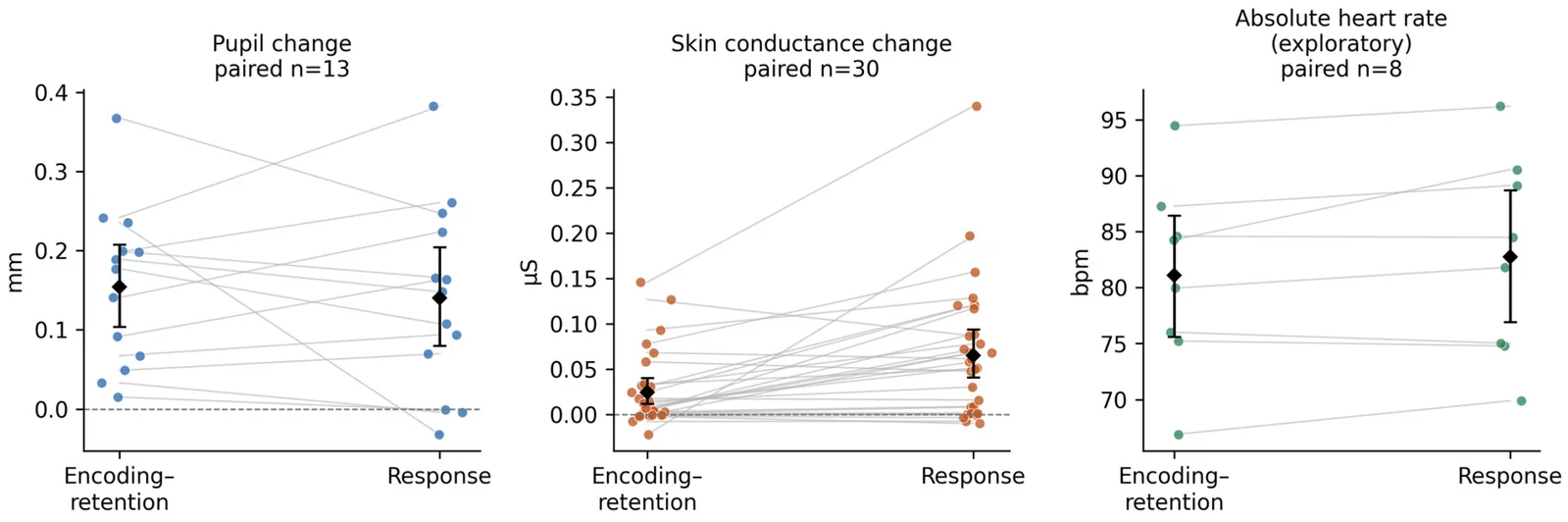
Participant-level stage features. Note. Points are participant means, gray lines connect paired participants, diamonds are group means, and error bars are bootstrap 95% confidence intervals. The heart-rate panel shows absolute descriptive values only. Its eight paired participants do not support a baseline-corrected HR comparison or a physiological conclusion about the stage difference.

As shown in Figure 3, pupil diameter increased relative to the pre-speech reference during encoding–retention (mean change = 0.17 mm, 95% CI [0.13, 0.21], Hedges’ gz = 1.84, n = 19, *q* < 0.001) and verbal response (0.14 mm, CI [0.08, 0.20], gz = 1.10, n = 13, *q* = 0.002). Among the 13 participants eligible for the paired contrast, the stage difference was uncertain (response minus encoding–retention = −0.01 mm, CI [−0.07, 0.04], gz = −0.12, *q* = 0.679). Stage-level skin conductance also increased during encoding–retention (0.02 μS, CI [0.01, 0.04], gz = 0.59, n = 30, *q* < 0.001) and verbal response (0.07 μS, CI [0.04, 0.09], gz = 0.85, n = 30, *q* < 0.001). Response exceeded encoding–retention by 0.04 μS (CI [0.02, 0.06], gz = 0.68, *q* < 0.001). This contrast describes task epochs and cannot isolate listening-related activity from articulation, respiration, movement, or delayed activity. Exploratory absolute mean HR was 79.94 bpm (SD = 11.09, n = 14) during encoding–retention and 80.34 bpm (SD = 11.20, n = 14) during response from eight participants.

**Figure 4:**
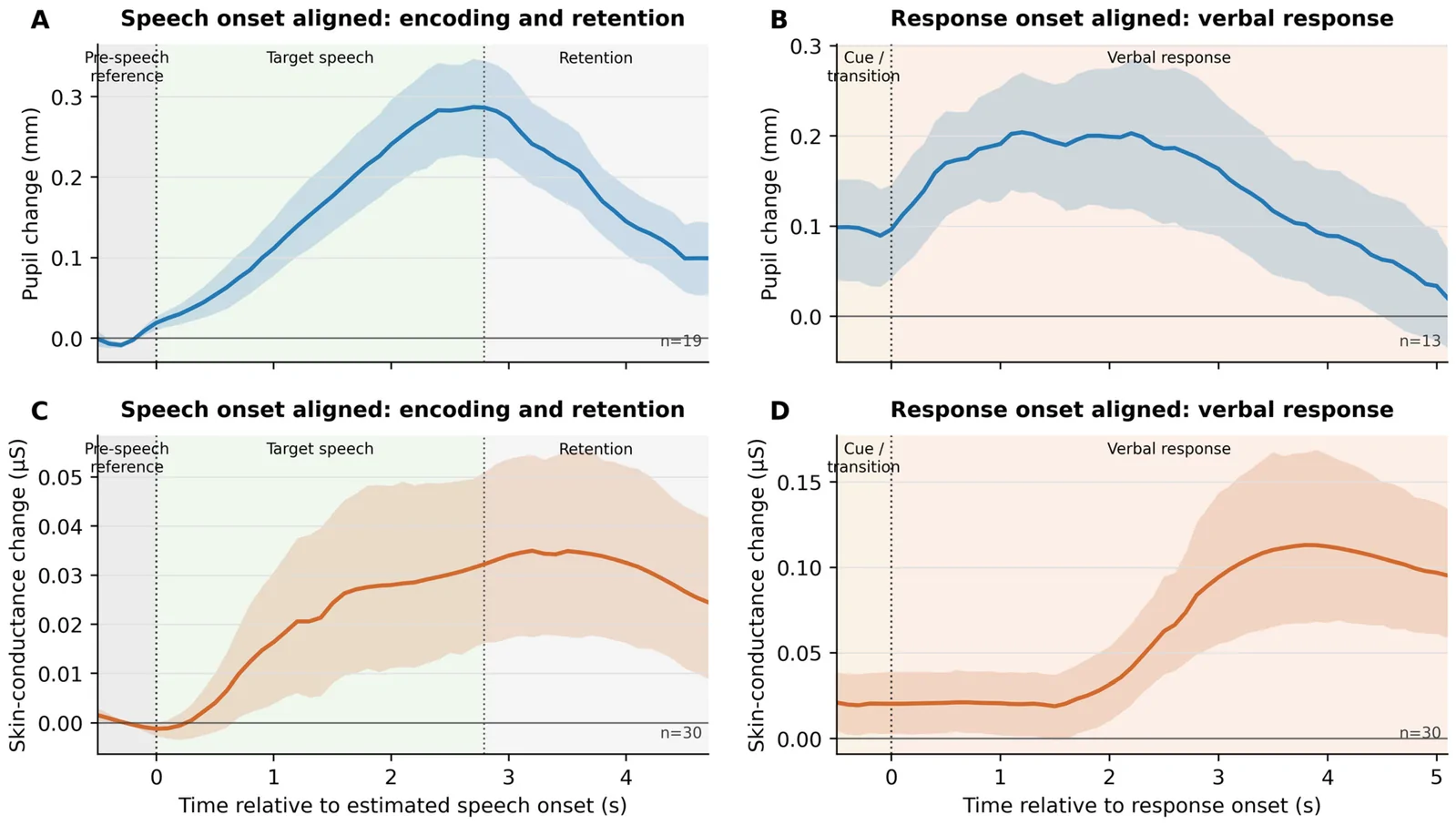
Descriptive group-level pupil and skin-conductance time courses. (**A**) Pupil change during encoding and retention, aligned to estimated target-speech onset (n = 19). (**B**) Pupil change during verbal response, aligned to response onset (n = 13). (**C**) Skin-conductance change during encoding and retention, aligned to estimated target-speech onset (n = 30). (**D**) Skin-conductance change during verbal response, aligned to response onset (n = 30). Note. Lines show group means of participant-average curves in native units, and shaded regions show 95% percentile bootstrap confidence intervals across participants. n denotes eligible participants, each with at least 14 valid trials for that modality and stage. The pre-speech reference is the 500 ms right before estimated target-speech onset during babble. Curves do not identify independent event-specific responses; EDA may include delayed activity from preceding events or stages. Heart rate is omitted because the 500 ms reference did not support valid reference-corrected HR estimates.

Figure 4 illustrates the temporal changes of pupil size and skin conduction during the sentence recognition task during two stages averaged across all valid participant trials. Pupil and skin-conductance changes were not significantly associated during encoding–retention (*ρ* = −0.25, n = 17, 95% CI [−0.67, 0.28], *p* = 0.34, *q* = 0.69) or response (*ρ* = 0.05, n = 12, [−0.60, 0.67], *p* = 0.89, *q* = 0.89). The intervals include both negative and positive associations. The data therefore provide no stable evidence of association in this sample and do not demonstrate independence.

### 3.3. Physiology–Behavior Associations

None of the 12 prespecified associations between pupil or skin-conductance stage means and SNR50, whole-sentence accuracy, or 15iSSQ survived FDR correction (Table 2). The pupil verbal-response association was the only nominal *p* < 0.05 result (*ρ* = 0.56, n = 13, 95% CI [0.08, 0.86], *p* = 0.05), but its *q* value was 0.58. Therefore, it is not interpreted as a positive finding. The small samples, especially for pupil outcomes, leave moderate associations unresolved.

### 3.4. Trial-Order Diagnostics

Mean pre-speech pupil diameter declined across trials (mean participant slope = −0.016 mm/trial, 95% CI [−0.021, −0.011], *q* < 0.001). Mean pupil changes during encoding–retention and response did not show clear trial-order slopes after correction. The skin-conductance reference showed no clear linear slope, whereas stage-level skin-conductance changes declined during encoding–retention (−0.006 μS/trial, [−0.010, −0.003], *q* < 0.001) and verbal response (−0.010 μS/trial, [−0.019, −0.004], *q* < 0.001). These trends are compatible with several time-on-task processes (i.e., habituation, fatigue); they do not identify a single cause or demonstrate recovery between trials.

## 4. Discussion

### 4.1. Main Findings

The design used individual speech-in-noise calibration to approximate a shared recognition level across listeners rather than testing everyone at the same SNR. But later whole-sentence accuracy averaged 43.6% and ranged from 20% to 70%. Thus, the physiological experiment tested participants near individually estimated psychometric midpoints rather than under verified equivalent-intelligibility conditions. Pupil diameter and stage-level skin conductance increased relative to a short local pre-speech reference. The paired pupil stage contrast was uncertain. Skin conductance was higher during verbal response, but that window combined articulation, respiration, movement, performance monitoring, and delayed activity from earlier events. The strongest supported interpretation is that skin conductance differed between task epochs, not that response required greater cognitive or listening effort.

### 4.2. Cross-Modal Interpretation

A comparison of sensor sensitivity would require the same participants measured across at least two validated demand levels and a defined criterion such as within-person slope, reliability-adjusted discrimination, or prediction of an independent effort rating. This study has one calibrated condition, unequal retention across channels, different physiological units and time constants, and no task-specific effort criterion. It can report native-unit task-stage effects and sensor feasibility but cannot rank modalities by sensitivity to listening demand. Likewise, absent or uncertain pupil–skin-conductance correlations are not evidence of independence: their CIs were wide, especially during response (n = 12), and included effects in both directions. Prior multichannel studies also show measure-specific patterns rather than a universal ranking [21,27,28].

### 4.3. Behavioral and Self-Report Findings

Whole-sentence accuracy varied from 20% to 70%, so calibration did not place participants at one common observed performance level, although 20 of 33 were within 40–60%. Individualization may have reduced performance heterogeneity relative to a fixed-SNR design, but this study did not include a fixed-SNR counterfactual and cannot estimate that reduction. Nor does the same recognition probability imply the same task demand or effort: listeners may invest different resources to reach similar accuracy [21,23]. The uncertain positive SNR50–accuracy trend is compatible with the formal task being easier for participants assigned higher, more favorable SNRs. The negative SNR50–SSQ trend was directionally consistent with better thresholds accompanying better self-reported hearing, but neither trend survived FDR and both CIs included zero.

No stage-mean physiology–behavior association survived correction, and directions were not uniform across modalities or stages. SNR50 represents an estimated speech-in-noise threshold and simultaneously determined the acoustic level used in the task for most participants; its association with a physiological outcome cannot by itself separate hearing ability from the effect of presented SNR. Whole-sentence accuracy is a noisy, 20-trial outcome in the later task, whereas 15iSSQ describes everyday hearing rather than in-task effort. These are complementary variables, not interchangeable adjustment factors or independent measures of hearing ability [9,10,11,19,21]. The nominal pupil verbal-response–SSQ correlation involved 13 participants, had *q* = 0.582, and may reflect response production or selection effects. Nonsignificant correlations cannot establish absent or independent physiological relationships in these samples.

The Appendix A audit further limits interpretation of SNR50 as a precisely measured individual-difference variable. Twenty adaptive trials left a mean posterior SD of 1.85 dB and an average conditional 95% credible-interval width of 7.25 dB; estimates still changed by 0.91 dB on average between trials 15 and 20. Results were also sensitive to the unvalidated fixed psychometric slope, with the shallower-slope replay shifting some estimates by up to 1.89 dB. Such measurement error can attenuate correlations, if they exist, with physiological outcomes and 15iSSQ.

### 4.4. Timing, Reference, and Carry-Over

The final 500 ms of the approximately 1 s babble-only interval supplied a local pre-speech reference, not a resting baseline. It follows babble onset by only 0.5 s and may contain orienting, anticipation, or activity from earlier trials [13,34,35]. After most verbal responses, only approximately 2–3 s without speech remained before the next trial; no recovery criterion or jitter was used. Figure 4 depicts descriptive group trajectories, not a sequence of independent event-specific responses. EDA evolves over several seconds, so a stage value may partly reflect preceding events or stages [15]. The declining pupil reference and skin-conductance changes across trials indicate time-on-task effects but do not prove carry-over in each trial or distinguish adaptation, habituation, fatigue, and baseline drift. Stage effects should be interpreted relative to this reference and pacing.

### 4.5. Cardiovascular Measurement

The 500 ms reference seldom contained a complete accepted pulse interval, leaving only two valid HR change trials among 660 encoding–retention trials and none during response. No participant reached the 14-trial threshold for baseline-corrected HR. Short epochs, ear-clip PPG artifacts, speaking, respiration, and movement further limit attribution [16]. Therefore, the temporal design was unsuitable for reliable reference-corrected PPG-derived HR estimation. Absolute HR values and the eight-person paired display in Figure 3 are retained solely to describe data availability; they support no stage-level physiological conclusion.

### 4.6. Strengths and Limitations

Strengths include simultaneous acquisition, an individual calibration intended to reduce fixed-SNR performance differences, use of event timestamps rather than planned timing, reconstruction of target-speech timing from code and stimulus files, modality-specific quality control, participant-level inference, and explicit uncertainty and multiplicity reporting.

There was no fixed-SNR comparison or second validated demand level, so this study cannot quantify any reduction in hearing-ability confounding or rank sensor sensitivity. Achieved accuracy varied, and equal accuracy would not prove equal effort. Encoding–retention included memory maintenance, while response included articulation, respiration, movement, performance monitoring, and delayed physiology. Without listening-only and matched speech-production controls, neither stage isolates listening effort. The short inter-trial interval could allow slow autonomic responses to overlap, but recovery was not measured. The software timing reconstruction lacked acoustic trigger validation. The 20-trial SNR50 procedure used a fixed, unvalidated slope and no precision stopping rule. No a priori power analysis determined the sample; the achieved-sample sensitivity limits leave small-to-moderate associations unresolved.

Pupil data loss was substantial: 19 participants were retained for encoding–retention and only 13 for verbal response, compared with 30 for each EDA stage. This limits precision and may bias the participant-level estimates if usable pupil data are related to participant characteristics. Descriptive retained-versus-excluded comparisons are reported in Appendix B; small samples do not permit a strong selection-bias test. The 15iSSQ was measured days to months before laboratory testing and was not a task-effort criterion. Other unmeasured factors include luminance, humidity, electrode contact area, respiration, and recovery between trials. The multiplicity families and retention criteria were defined during peer-review reanalysis rather than prospectively preregistered. The young volunteer sample and controlled acoustic setting limit generalizability.

### 4.7. Clinical Relevance and Future Work

Speech-recognition scores and general hearing questionnaires do not directly measure resources invested during a specific listening task. Physiological recording might eventually add useful information to audiological assessment, but this study does not establish clinical validity, incremental value, effort specificity, independence among sensors, or comparative sensitivity to changing demand. The present contribution is methodological: pupil and skin-conductance responses were measurable in subsets of participants at one condition, whereas the short PPG reference did not support HR change estimation.

A direct test of the design premise should compare fixed-SNR and individually calibrated designs, quantify how each affects the spread of achieved accuracy and residual hearing-ability associations, and include at least two or three individualized intelligibility levels, such as 50% and 90%. A prespecified within-person demand slope, test–retest reliability, and prediction of concurrent effort ratings or another independent criterion could then support a fair sensor-sensitivity comparison. Listening-only and matched verbal-speech conditions would help separate encoding from articulation. Longer intertrial intervals with an explicit recovery criterion would reduce overlap; if faster pacing is necessary, continuous EDA response modeling or deconvolution could be evaluated, provided its assumptions and event timing are validated [15]. Electrocardiogram or validated PPG with longer, well-supported epochs is needed for cardiac inference. Future calibration should justify or estimate the psychometric slope, continue to a precision criterion, and include independent fixed-level validation. Larger samples that allow for sensor loss, including older adults and people with hearing loss, are needed before clinical generalization or longitudinal rehabilitation applications.

## 5. Conclusions

Individual calibration supplied an approximate recognition anchor, but later whole-sentence accuracy remained variable. Pupil diameter and stage-level skin conductance increased relative to a short pre-speech reference during sentence recognition in babble. Skin conductance differed between encoding–retention and verbal response, whereas the paired pupil contrast was uncertain. No cross-modal or physiology–behavior association survived multiplicity correction. The short reference did not support baseline-corrected HR. These findings characterize task-epoch reactivity and sensor feasibility at one participant-specific SNR condition. They do not establish equivalent intelligibility or effort, removal of hearing-ability confounding, or comparative sensitivity to listening demand.

## Figures and Tables

**Table 1:** Main subjective, behavioral and physiological measures used in listening-effort research.

Category	Specific Metrics	Mechanism	Advantages	Limitations
Subjective	Questionnaires and rating scales (e.g., NASA Task Load Index [18])	Conscious appraisal of cognitive exertion and task demand [9]	High face validity and clinical accessibility [19] Captures the listener’s subjective lived experience [9,20]	Vulnerable to response bias [21] Easily confounded by perceived task success or external variables rather than actual exertion [19,20]
Behavioral	Processing speed and dual-tasking (e.g., reaction time, accuracy) [19,22]	Allocation of limited cognitive capacity and divided attention [9,19]	High ecological validity for multitasking situations [19] Provides a direct, quantifiable behavioral cost of auditory masking [22]	Susceptible to practice effects and speed–accuracy tradeoffs [19,20] Insensitive at ceiling performance levels [19]
Physiological	Pupillometry (e.g., peak or mean pupil dilation, Pupil Unrest Index) [20,22]	Autonomic arousal and central cognitive load, driven by the locus coeruleus–norepinephrine system [21]	Continuous measurement with high temporal resolution [22] Sensitive to real-time changes in cognitive resource allocation and sustained fatigue [19,20]	Confounded by motor preparation and luminance [21,22] Exhibits a non-monotonic (inverted U-shape) function at extreme task difficulty [21]
	Electrodermal activity (e.g., skin conductance) [19,21]	Pure sympathetic nervous system arousal via eccrine gland activity [21]	Isolates sympathetic arousal without parasympathetic interference [21] Sensitive to the conscious expectation of poor performance [19]	Prone to habituation to repetitive stimuli [19]; low temporal resolution requiring minutes to stabilize [9,19] High inter-session variability limits longitudinal comparisons [19]
	Cardiovascular (e.g., heart rate) [21]	Regulated by both the sympathetic and parasympathetic branches of the autonomic nervous system [21]	Allows for branch-specific isolation, such as using pre-ejection period for sympathetic contractility and high-frequency heart rate variability for parasympathetic withdrawal [21]	Insensitive to minor or rapid shifts in task demand [21] Slow physiological latency, taking 10 to 20 s to reach a peak response [17]
	Central or cortical (e.g., electroencephalography, functional near-infrared spectroscopy) [9,21]	Direct cortical activity tracking working memory load and the active suppression of noise [9,21]	Provides direct neural correlates [21] Offers high spatial precision via functional near-infrared spectroscopy or high temporal precision via electroencephalography [21]	Inconsistent directionality of effects, such as alpha power increasing or decreasing across different masking paradigms [9,21] High susceptibility to electromagnetic interference between devices [17]

Note. These methods are used in listening-effort research but are not interchangeable or specific to listening effort.

**Table 2:** Associations between stage-mean physiological and behavioral measures.

Physiological Feature	Behavior	n	*ρ* [95% CI]	Raw *p*	BH *q*
Pupil encoding–retention mean	SNR50	19	−0.033 [−0.496, 0.454]	0.891	0.920
Pupil encoding–retention mean	Whole-sentence accuracy	19	−0.106 [−0.562, 0.425]	0.666	0.881
Pupil encoding–retention mean	15-item SSQ	19	0.246 [−0.213, 0.611]	0.307	0.870
Pupil verbal-response mean	SNR50	13	−0.033 [−0.672, 0.668]	0.920	0.920
Pupil verbal-response mean	Whole-sentence accuracy	13	−0.237 [−0.808, 0.461]	0.435	0.870
Pupil verbal-response mean	15i SSQ	13	0.562 [0.079, 0.860]	0.048	0.582
Skin conductance, encoding–retention	SNR50	30	0.067 [−0.329, 0.451]	0.724	0.881
Skin conductance, encoding–retention	Whole-sentence accuracy	30	0.185 [−0.210, 0.544]	0.325	0.870
Skin conductance, encoding–retention	15i SSQ	30	−0.097 [−0.454, 0.278]	0.610	0.881
Skin conductance, verbal response	SNR50	30	−0.159 [−0.536, 0.271]	0.398	0.870
Skin conductance, verbal response	Whole-sentence accuracy	30	0.249 [−0.132, 0.572]	0.183	0.870
Skin conductance, verbal response	15i SSQ	30	0.064 [−0.340, 0.456]	0.734	0.881

Note. Stage means are participant-level changes from the local pre-speech reference; q values use one 12-test Benjamini–Hochberg family defined during revision. SNR50 = model-based whole-sentence 50% SNR midpoint; 15iSSQ = 15-item Speech, Spatial and Qualities of Hearing Scale.

**Figure A1:**
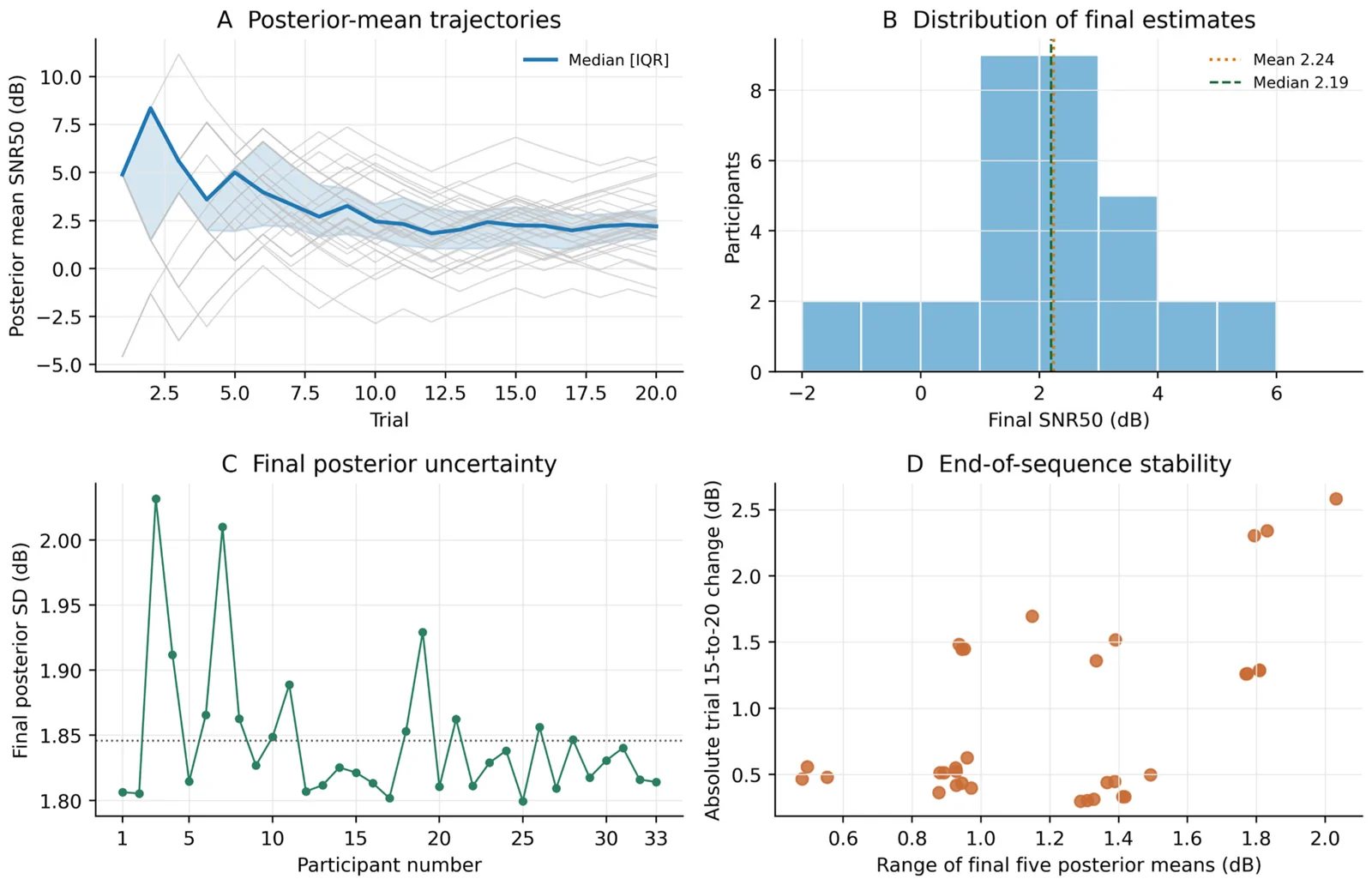
Calibration diagnostics of recorded SNR50 data. Note. (**A**) Individual posterior-mean trajectories with the across-participant median and IQR. (**B**) Distribution of final SNR50 estimates. (**C**) Final posterior SD for each participant. (**D**) End-of-sequence stability by comparing derived SNR values from trial 15 to trial 20. All panels include all 33 participants.

**Figure A2:**
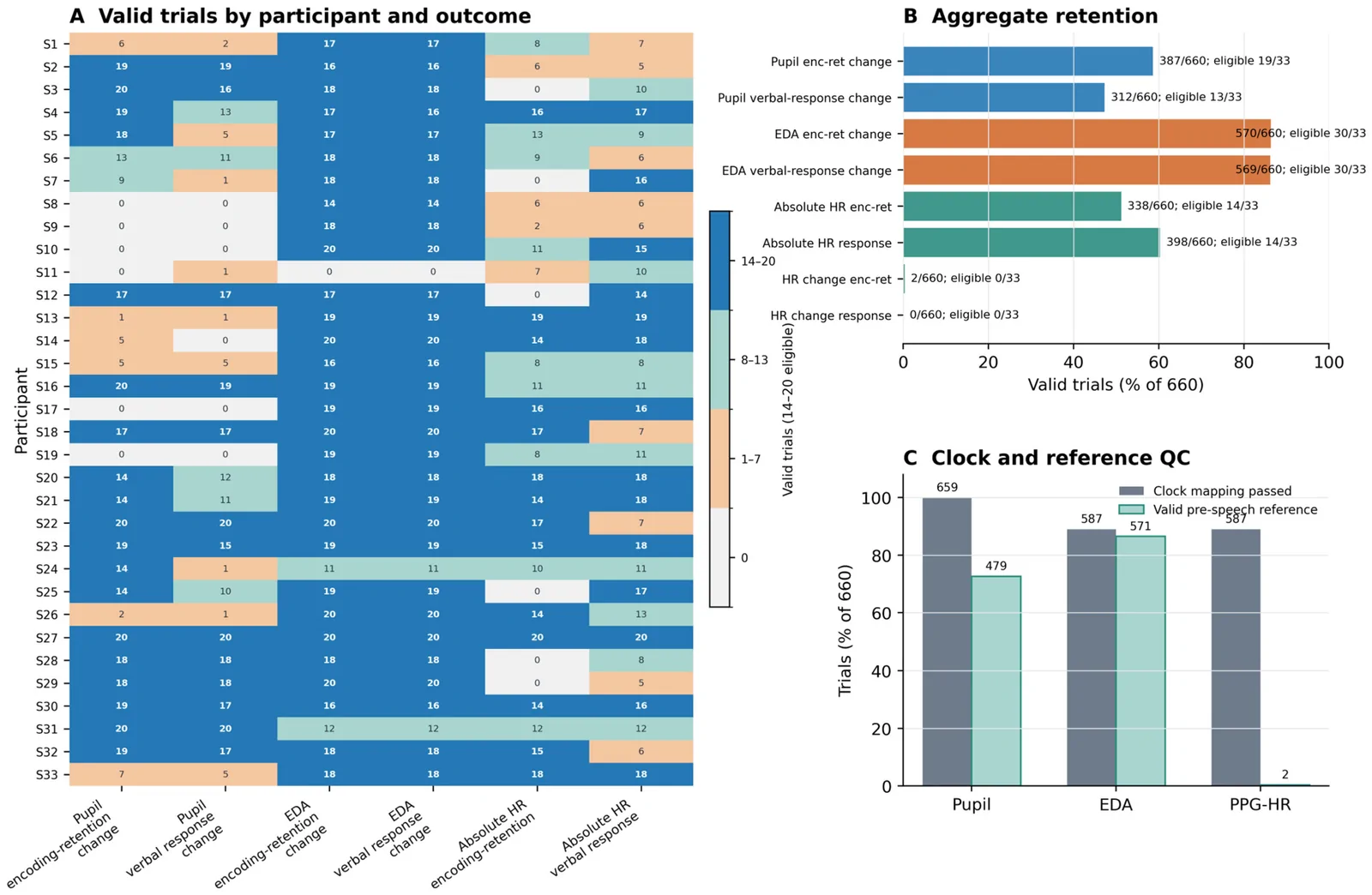
Physiological preprocessing quality control and data retention. Note. (**A**) Participant-level valid-trial counts; blue cells meet the 14-of-20 eligibility criterion. (**B**) Aggregate valid-trial percentages and numbers of eligible participants, including unsupported reference-corrected HR for completeness. (**C**) Clock-mapping pass rates and valid pre-speech-reference rates. Counts derive from the prespecified 0.5 s reference analysis. Eligibility was evaluated independently for each modality and stage.

## Data Availability

The data presented in this study are available on request from the corresponding author due to privacy concerns.

## Abbreviations

The following abbreviations are used in this manuscript:

15iSSQ	15-item Speech, Spatial, and Qualities of Hearing Scale
CI	Confidence Interval
EDA	Electrodermal Activity
FDR	False Discovery Rate
HINT	Hearing-in-Noise Test
HR	Heart Rate
PPG	Photoplethysmography
SNR	Signal to Noise Ratio

## Appendix A. Model-Based Whole-Sentence SNR50 Calibration

### Appendix A.1. Task and Scoring

Individualized SNRs were estimated from 20 HINT sentences [29], following previous laboratory practice. The experimenter marked a response correct (1) with the right-arrow key (→) only if the entire sentence was repeated correctly; any word error was marked incorrect (0) with the left-arrow key (←).

### Appendix A.2. Implemented Bayesian Model

For each trial, the implemented Bernoulli likelihood was defined by the logistic function,

$$P(Y=1\;|\;x,\;\theta )=\frac{1}{1+e^{\left[-\beta (x-\theta )\right]}}$$

where *x* is the presented SNR, *θ* is the model-based whole-sentence SNR50, and *β* was fixed at 0.25 dB^−1^. A Gaussian prior with mean 0 dB and SD 8 dB was normalized over a 300-point grid from −20 to +30 dB. After each binary response, the posterior was proportional to the preceding posterior multiplied by *P*(*Y* = 1 | *x*, *θ*) for a correct response or 1 − *P*(*Y* = 1 | *x*, *θ*) for an incorrect response. The posterior mean determined the next presentation SNR and the final calibration estimate after trial 20. At the midpoint, the assumed local slope is *β*/4 = 0.0625, or 6.25 percentage points per dB.

Although the intended SNR target was 75% recognition, that target probability was absent from the implemented likelihood and presentation rule. In the implemented model, *P*(*Y* = 1 | *x*, *θ*) = 0.50. We therefore call the estimate model-based whole-sentence SNR50. It is not a direct demonstration that later task accuracy equaled 50%.

### Appendix A.3. Individual Results and Audit

As shown in Figure A1, final posterior uncertainty remained substantial across 33 archived calibration histories: posterior SD averaged 1.85 dB (range 1.80–2.03), and the mean 95% credible-interval width was 7.25 dB. The range of the final five posterior means averaged 1.22 dB (range 0.48–2.03). The mean absolute change between the trial-15 and trial-20 estimates was 0.91 dB, with a maximum of 2.58 dB. Thus, completing 20 trials reduced uncertainty but did not demonstrate participant-level convergence.

The observations were replayed under the implemented model and four alternative specifications. The replay exactly reproduced the saved endpoints. Changing only the prior width had a modest average effect, whereas changing the unvalidated psychometric slope had a larger effect. A shallower slope (*β* = 0.15 dB^−1^) shifted posterior means by 0.60 dB on average in absolute value and by as much as 1.89 dB; it also increased mean posterior SD to 2.90 dB. A steeper slope (*β* = 0.35 dB^−1^) shifted means by 0.27 dB on average in absolute value and reduced mean posterior SD to 1.36 dB. These replays are conditional on the trials selected by the implemented model and do not reproduce the stimulus sequence that an alternative model would have selected prospectively.

**Table A1:** Sensitivity of the final posterior summary to the fixed slope and prior width.

Scenario	Specification	n	Mean Difference (dB)	Mean Absolute Difference (dB)	Maximum Absolute Difference (dB)	Mean Posterior SD (dB)
Implemented	*β* = 0.25; prior SD = 8	33	0.000	0.000	0.000	1.846
Shallower slope	*β* = 0.15; prior SD = 8	33	−0.379	0.603	1.894	2.901
Steeper slope	*β* = 0.35; prior SD = 8	33	0.142	0.272	0.874	1.358
Narrower prior	*β* = 0.25; prior SD = 4	33	−0.300	0.325	0.776	1.710
Wider prior	*β* = 0.25; prior SD = 16	33	0.091	0.099	0.236	1.886

Note. Differences are relative to the implemented *β* = 0.25, prior-mean = 0 dB, prior-SD = 8 dB model. Alternative models were replayed over the same observed, adaptively selected trials.

## Appendix B. Physiological Data Preprocessing

### Appendix B.1. Pupil Preprocessing and Feature Extraction

Each eye was processed independently. Samples were invalidated when Tobii validity was not 1, diameter was nonpositive or missing, or the sample fell within 100 ms of an invalid run. Remaining extreme values were removed when they exceeded 4.5 median-absolute-deviation scale units from the trial median. Only gaps no longer than 250 ms with observed samples on both sides were linearly interpolated; longer gaps and edge gaps remained missing. The reference for each eligible eye was the median of cleaned, genuinely observed samples in the pre-speech interval. At least 70% observed reference coverage was required. Subtractive correction in millimeters was applied separately to each eye, after which eyes were averaged. Observed samples were preferred over contralateral interpolated samples. Stage features were time-weighted mean changes from reference. Display smoothing was not used for feature extraction [34,35].

### Appendix B.2. Electrodermal Preprocessing and Feature Extraction

Samples with invalid codes, conversion mismatch, conductance outside 0.2–125 μS, or the 80 ms settling interval after an automatic range change were removed. A second-order 5 Hz low-pass filter was applied only within continuous valid segments; 100 ms at each filtered segment boundary were discarded, and gaps were not bridged. The primary electrodermal outcome was the time-weighted mean skin conductance within each stage minus the mean pre-speech reference. Absolute mean conductance was retained descriptively. Because the alert tone, babble onset, speech, response cue, and verbal response occurred at short intervals, activity could overlap across stages. The result is therefore described as stage-level skin-conductance change, not an isolated speech-evoked skin conductance response [15,16].

### Appendix B.3. PPG-Derived Heart Rate

PPG was band-pass filtered from 0.5 to 5 Hz within continuous nonzero segments. Candidate pulse intervals were detected from peaks, restricted to 40–140 bpm, and required a correlation of at least 0.70 with the participant-trial median pulse template. Heart rate was defined only inside accepted adjacent pulse intervals; rejected intervals and recording edges remained missing. The 500 ms reference rarely contained a complete supported pulse interval: only two of 660 encoding–retention trials and no response trials yielded a valid change score, and no participant reached the 14-trial threshold. Baseline-corrected heart rate, short-window heart rate variability, and heart-rate peak latency were therefore excluded from primary inference. Absolute mean heart rate was retained only as an exploratory data-availability description [16].

### Appendix B.4. Quality-Control Results

Clock mapping passed for 659 of 660 gaze trial files and 587 of 660 Shimmer trial files. Among the 73 rejected Shimmer files, 29 contained duplicate or reversed device-clock ticks and 44 failed the mapping-residual criterion. Because EDA and PPG shared the Shimmer device clock, these failures excluded both streams for the affected trial. Clock alignment was derived from software event and reception times and was not independently verified by a hardware trigger.

A valid pre-speech reference was available in 479 of 660 pupil trials (72.6%), 571 EDA trials (86.5%), and 2 PPG-derived HR trials (0.3%). As shown in Table A2, the pupil analysis retained 387 trials and 19 eligible participants during encoding–retention and 312 trials and 13 eligible participants during verbal response. EDA retained 570 trials and 30 eligible participants during encoding–retention and 569 trials and 30 eligible participants during verbal response. No participant met the HR change-score threshold. Exploratory absolute HR, which did not require reference support, retained 338 encoding–retention trials and 398 response trials, with 14 eligible participants in each stage; only eight were eligible for both stages.

**Table A2:** Trial retention and participant eligibility after physiological preprocessing.

Outcome	Stage	Recorded n	Valid Trials	Retained n	Dominant Trial-Level Limitation
Pupil change	Encoding–retention	33	387/660 (58.6%)	19	Reference unavailable (180 trials); low stage support (92)
Pupil change	Verbal response	33	312/660 (47.3%)	13	Reference unavailable (180); low stage support (167)
Skin conductance	Encoding–retention	33	570/660 (86.4%)	30	Shimmer clock QC (73); low support (17)
Skin conductance	Verbal response	33	569/660 (86.2%)	30	Shimmer clock QC (73); low support (18)
Absolute HR	Encoding–retention	33	338/660 (51.2%)	14	Insufficient accepted pulse support (249); clock QC (73)
Absolute HR	Verbal response	33	398/660 (60.3%)	14	Insufficient accepted pulse support (189); clock QC (73)
HR change	Encoding–retention	33	2/660 (0.3%)	0	500 ms reference without supported pulse interval
HR change	Verbal response	33	0/660 (0.0%)	0	500 ms reference without supported pulse interval

Note. Retained means at least 14 of 20 valid trials for that outcome and stage. Loss categories are trial-level accounting, not mutually exclusive participant exclusion diagnoses. Absolute HR was descriptive; eight participants had eligible absolute HR in both stages.

Missingness was concentrated in particular participant-by-modality combinations rather than being uniform across the sample (Figure A2A). Consequently, a single cross-sensor complete-case sample would have discarded usable data and was not imposed. To assess possible selection, we compared baseline behavioral descriptors without significance testing. For pupil encoding–retention, retained versus excluded participants had mean SNR50 of 2.45 versus 1.95 dB and later accuracy of 45.8% versus 40.7% (n = 19 versus 14). For pupil response, the corresponding values were 2.27 versus 2.22 dB and 45.0% versus 42.8% (n = 13 versus 20). These descriptive differences cannot establish comparability or rule out selection bias.
